# Effect of vessel enhancement filters on the repeatability of measurements obtained from widefield swept-source optical coherence tomography angiography

**DOI:** 10.1038/s41598-020-79281-3

**Published:** 2020-12-17

**Authors:** Jimmy Hong, Mengyuan Ke, Bingyao Tan, Amanda Lau, Damon Wong, Xinwen Yao, Xinyu Liu, Leopold Schmetterer, Jacqueline Chua

**Affiliations:** 1 Singapore Eye Research Institute, Singapore National Eye Centre, 20 College Road, The Academia, Level 6, Discovery Tower, Singapore, 169856 Singapore; 2 Department of Internal Medicine, Singapore General Hospital, Singapore, Singapore; 3 SERI-NTU Advanced Ocular Engineering (STANCE), Singapore, Singapore; 4 Institute of Health Technologies, Nanyang Technological University, Singapore, Singapore; 5 Ophthalmology and Visual Sciences Academic Clinical Program, Duke-NUS Medical School, National University of Singapore, Singapore, Singapore; 6 Department of Clinical Pharmacology, Medical University of Vienna, Vienna, Austria; 7 Center for Medical Physics and Biomedical Engineering, Medical University of Vienna, Vienna, Austria; 8 Institute of Molecular and Clinical Ophthalmology, Basel, Switzerland

**Keywords:** Optical imaging, Tomography, Eye diseases

## Abstract

We assessed the inter-visit repeatability of 15 × 9-mm^2^ swept-source OCTA (SS-OCTA; PLEX Elite 9000, Carl Zeiss Meditec) metrics in 14 healthy participants. We analysed the perfusion density (PD) of large vessels, superficial capillary plexus (SCP), and deep capillary plexus (DCP) as well as choriocapillaris flow voids in 2 different regions: the macular region and peripheral region. Also, retinal plexus metrics were processed further using different filters (Hessian, Gabor and Bayesian) while choriocapillaris flow voids were calculated with 1 and 1.25 standard deviation (SD) thresholding algorithms. We found excellent repeatability in the perfusion densities of large vessels (ICC > 0.96). Perfusion densities varied with different filters in the macular region (SCP: 24.12–38.57% and DCP: 25.16–38.50%) and peripheral (SCP: 30.52–39.84% and DCP: 34.19–41.60%) regions. The ICCs were lower in the macular region compared to the peripheral region and lower for DCP than for SCP. For choriocapillaris flow voids, the 1.25 SD threshold resulted in fewer flow voids, while a good ICC (ICC > 0.81) was achieved using either threshold settings for flow void features in both regions. Our results suggest good repeatability of widefield SS-OCTA for the measurements of retinal perfusion density and choriocapillaris flow voids, but measurements from different filters should not be interchanged.

## Introduction

Changes in the peripheral vasculature of the retinal and choriocapillaris layer have been implicated in a myriad of ocular diseases. These vascular changes vary in topographical distribution based on the underlying disease, such as the presence of non-perfusion areas in diabetic retinopathy (DR)^1–8^ and branch retinal vein occlusion^9^, neovascularisation in proliferative DR^10^ and ischaemic retinal vein occlusion^11^, and choriocapillaris perfusion flow deficits in acute syphilitic placoid chorioretinitis^12^. The advent of swept-source optical coherence tomography angiography (SS-OCTA) technology has enabled high-definition widefield images that visualise the retinal and choriocapillaris vasculature in one single scan containing volumetric data^13^. For the widefield SS-OCTA technology to be of clinical utility, it must provide repeatable measurements.

Earlier studies have shown good intra-visit repeatability of retinal density measurements using a single 12 × 12-mm^2^ OCTA scan^14^ and moderate inter-visit repeatability using montaged 12 × 12-mm^2^ OCTA scans^15^. Recent advancements in OCTA technology have allowed for an even wider field OCTA imaging, with 15 × 9-mm^2^ scans of the posterior pole using the PLEX Elite 9000 (Carl Zeiss Meditec, Inc.), capturing different regions of the retinal capillary plexus and choriocapillaris. However, the novel 15 × 9-mm^2^ scan protocol has several unique challenges and its repeatability has yet to be assessed. Widefield scans suffer from higher occurrences of low-OCT-signal artefacts in the peripheral region, which are known as vignetting^16^. It remains unclear if the repeatability of widefield scans is comparable between the macular and peripheral regions. Moreover, studies have used different vessel enhancement filters for the depiction and calculation of the angiograms, such as Hessian filter^17,18^ and Gabor filter^19,20^. Although studies have often reported good repeatability using these filters, it is important to take note that the use of different filters have not been assessed on the same scan.

Through this study, we aim to study the inter-visit repeatability of this new 15 × 9-mm^2^ widefield scan protocol in healthy individuals by comparing the repeatability of metrics between the macular and peripheral regions, and studying the impact of different vessel enhancement filters on the repeatability metrics. A highly repeatable widefield SS-OCTA technology is imperative for assessing the significance of differences that may be seen over time or between normal and diseased eyes.

## Results

### Patient demographics and ocular data

Out of the 15 healthy individuals, one individual was excluded because of poor image quality. A total of 14 eyes from 14 healthy individuals were included. Their mean ± SD age was 31 ± 6 years old, and 5 (36%) were male. All study participants were Asian, of which 11 were Chinese and 3 were Indian. Of the 14 eyes, 12 (86%) were right eyes. Study eyes had a spherical equivalent of − 3.97 ± 2.95 D, and an axial length of 24.79 ± 1.57 mm. The OCTA scans had comparable signal strength indices between the first (9.93 ± 0.27) and second visits (9.79 ± 0.43; P = 0.165). There was no difference in the OCTA measurements between right eye versus left eye or between Chinese and Indian participants (all P > 0.05).

### Large retinal vessels

**Figure 1:**
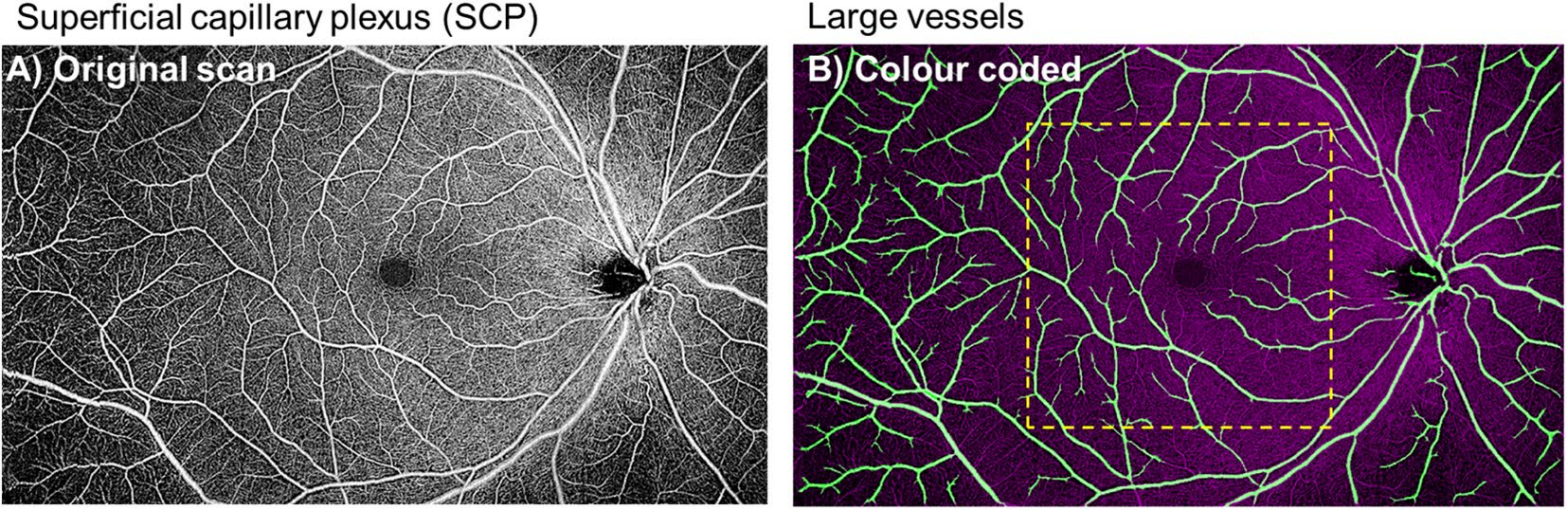
The superficial capillary plexus image (**A**) was processed to obtain the large vessel metrics (**B**; colored coded in green). Perfusion densities were investigated in 2 different regions of the large vessels: (1) the macular region (within yellow dotted box) with a 6 × 6-mm^2^ region centred over the fovea and (2) the peripheral region (outside the yellow dotted box). Image A was generated from the built-in review software (PLEX Elite Review Software, Carl Zeiss Meditec, Inc., Dublin, USA; Version 1.7.1.31492; https://www.zeiss.fr/content/dam/Meditec/international/ifu/documents/plex-elite/current/2660021169042_rev._a_artwork.pdf).

The PD for large vessels of the two visits is shown in Table 1 and Fig. 1. There were no significant differences in the large vessel measurements between visits for both analysed regions (P = 0.540 for the macular region [ICC = 0.96; 95% limits of agreement, − 0.65% to 0.80%], and P = 0.360 for the peripheral region [ICC = 0.97; 95% limits of agreement, − 0.53% to 0.42%], respectively), indicating excellent repeatability. The mean differences in macular and peripheral large vessels PD between visits were 0.07% and − 0.06%, respectively (Fig. 4A,B). Furthermore, the spread of the differences remained consistent across the range of PD measurements.

**Table 1:** OCTA vascular parameters of the retinal plexus.

Characteristics	(A) Averaged of 2 visits Mean (SD)	P value of paired t-test	(B) ICC (95% CI)	(C) Pearson’s *r* (95% CI)	P value
**Perfusion density large vessels (%)**					
Macula	7.72 (0.82)	0.540	**0.96 (0.87, 0.99)**	NA	NA
Periphery	9.74 (0.63)	0.360	**0.97 (0.90, 0.99)**	NA	NA
**Superficial perfusion density (%)**					
Macula					
1. Original scan	38.57 (0.74)	0.512	**0.87 (0.59, 0.96)**	Reference	
2. Hessian filter	36.50 (0.82)	0.330	**0.90 (0.71, 0.97)**	**0.83 (0.53, 0.94)**	< 0.001
3. Gabor filter	24.12 (0.94)	0.870	**0.90 (0.68, 0.97)**	**0.87 (0.62, 0.96)**	< 0.001
4. Bayesian filter	33.49 (0.93)	0.819	0.72 (0.10, 0.91)	**0.92 (0.77, 0.98)**	< 0.001
Periphery					
1. Original scan	39.84 (0.77)	0.765	**0.92 (0.75, 0.97)**	Reference	
2. Hessian filter	38.13 (1.04)	0.959	**0.96 (0.87, 0.99)**	**0.79 (0.44, 0.93)**	< 0.001
3. Gabor filter	30.52 (1.00)	0.656	**0.95 (0.86, 0.99)**	**0.93 (0.78, 0.98)**	< 0.001
4. Bayesian filter	34.93 (1.03)	0.524	**0.85 (0.55, 0.95)**	**0.84 (0.57, 0.95)**	< 0.001
**Deep perfusion density (%)**					
Macula					
1. Original scan	38.50 (0.70)	0.435	**0.77 (0.31, 0.93)**	Reference	
2. Hessian filter	33.24 (0.54)	0.209	**0.78 (0.34, 0.93)**	**0.66 (0.19, 0.88)**	0.011
3. Gabor filter	25.16 (1.48)	0.848	0.70 (0.03, 0.91)	**0.79 (0.44, 0.93)**	< 0.001
4. Bayesian filter	30.83 (1.37)	0.389	0.21 (0, 0.75)	**0.77 (0.41, 0.93)**	0.001
Periphery					
1. Original scan	41.60 (0.71)	0.456	**0.84 (0.50, 0.95)**	Reference	
2. Hessian filter	36.09 (0.99)	0.325	**0.83 (0.50, 0.95)**	**0.77 (0.41, 0.93)**	0.001
3. Gabor filter	34.19 (0.74)	0.252	**0.88 (0.65, 0.96)**	0.48 (-0.07, 0.81)	0.081
4. Bayesian filter	34.51 (1.18)	0.071	**0.78 (0.33, 0.93)**	**0.70 (0.27, 0.90)**	0.005

Bold interface indicates the filter that provided good ICC (≥ 0.75) and P < 0.05 for Pearson’s correlation when compared to the original scan for each category.

*SD* standard deviation; *CI* confidence interval; *ICC* intraclass correlation coefficient.

### Superficial perfusion density

**Figure 2:**
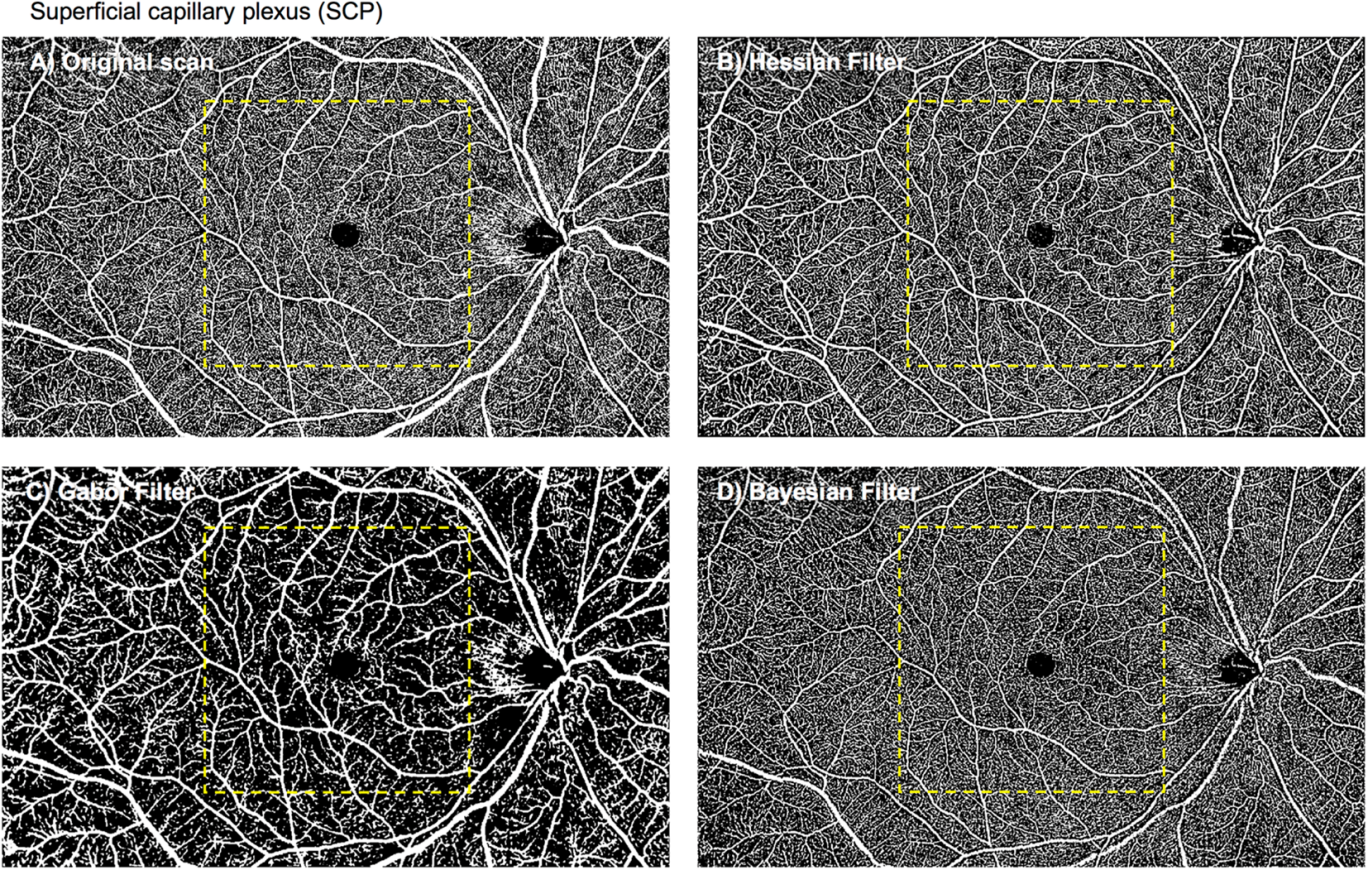
Superficial capillary plexus: original scan (**A**) and enhanced with various filters (**B**–**D**). Perfusion densities were investigated in 2 different regions of the superficial capillary plexus: (1) the macular region (within yellow dotted box) with a 6 × 6-mm^2^ region centred over the fovea and (2) the peripheral region (outside the yellow dotted box).

Regarding the superficial perfusion density obtained without any filter (termed as the original scan; Table 1 and Fig. 2), there were no significant differences between visits in both the analysed regions (P = 0.512 for the macular region [ICC = 0.87; 95% limits of agreement, − 0.96% to 1.19%], and P = 0.765 for the peripheral region [ICC = 0.92; 95% limits of agreement, − 0.82% to 0.95%], respectively), suggesting an overall good to excellent repeatability. The mean differences in macular and peripheral superficial PD between visits were 0.11% and − 0.07%, respectively (Fig. 4C,D). The spread of the differences remained consistent across the range of PD measurements.

The 3 filters presented with different measurement values, in both the regions (24.12 to 36.50% for the macular region; 30.52 to 38.13%; both P < 0.001). The ICC with the vessel enhancement filters for both regions remained good (ICC = 0.90 for the macular region and ICC = 0.85 to 0.96 for the peripheral region), except with the Bayesian filter for the macular region (ICC = 0.72; Table 1). All 3 filters exhibited good correlation with the original scan for both macular (r = 0.83 to 0.92) and peripheral regions (r = 0.79 to 0.93).

### Deep perfusion density

**Figure 3:**
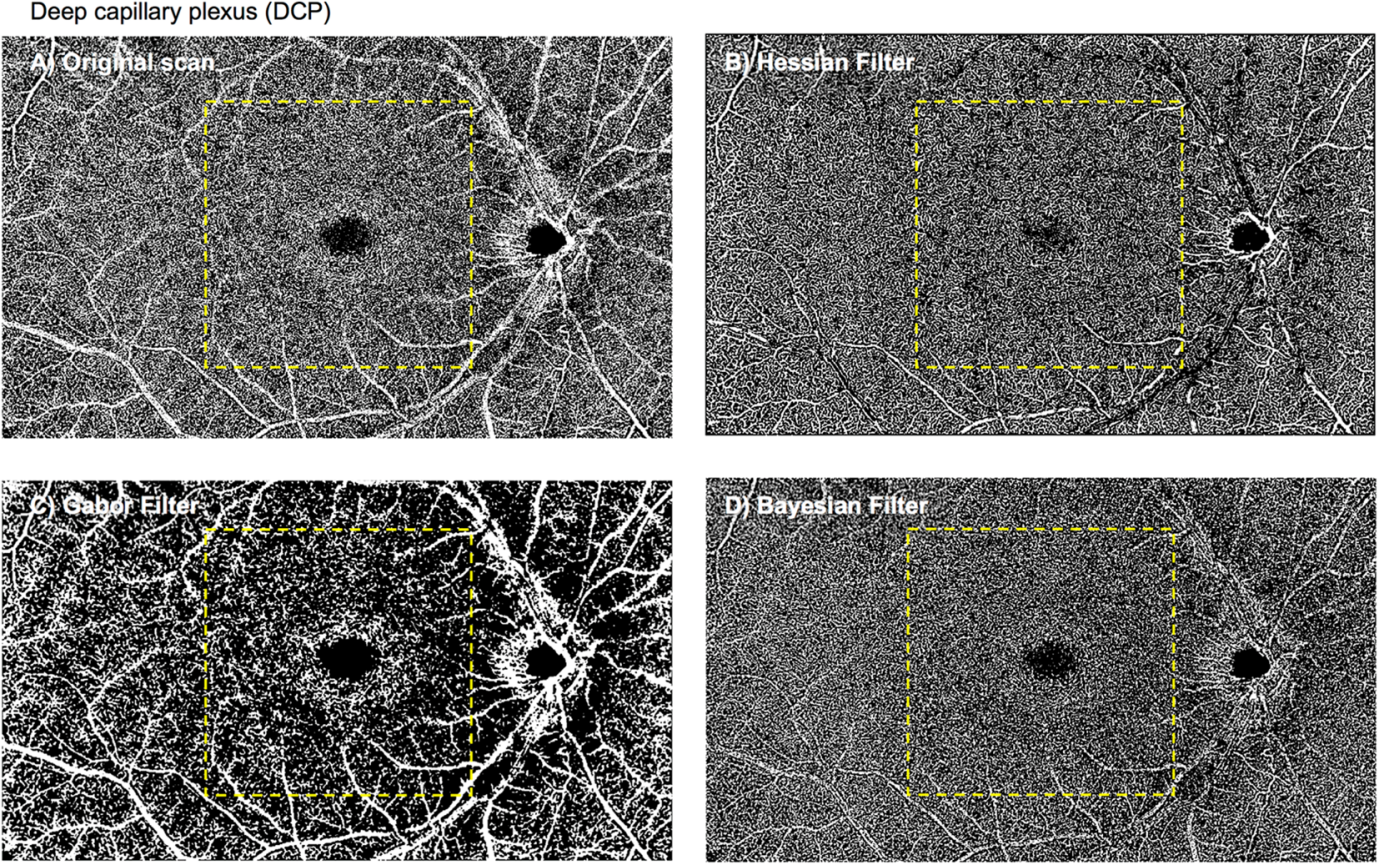
Deep capillary plexus: original scan (**A**) and enhanced with various filters (**B**–**D**). Perfusion densities were investigated in 2 different regions of the deep capillary plexus: (1) the macular region (within yellow dotted box) with a 6 × 6-mm^2^ region centred over the fovea and (2) the peripheral region (outside the yellow dotted box).

**Figure 4:**
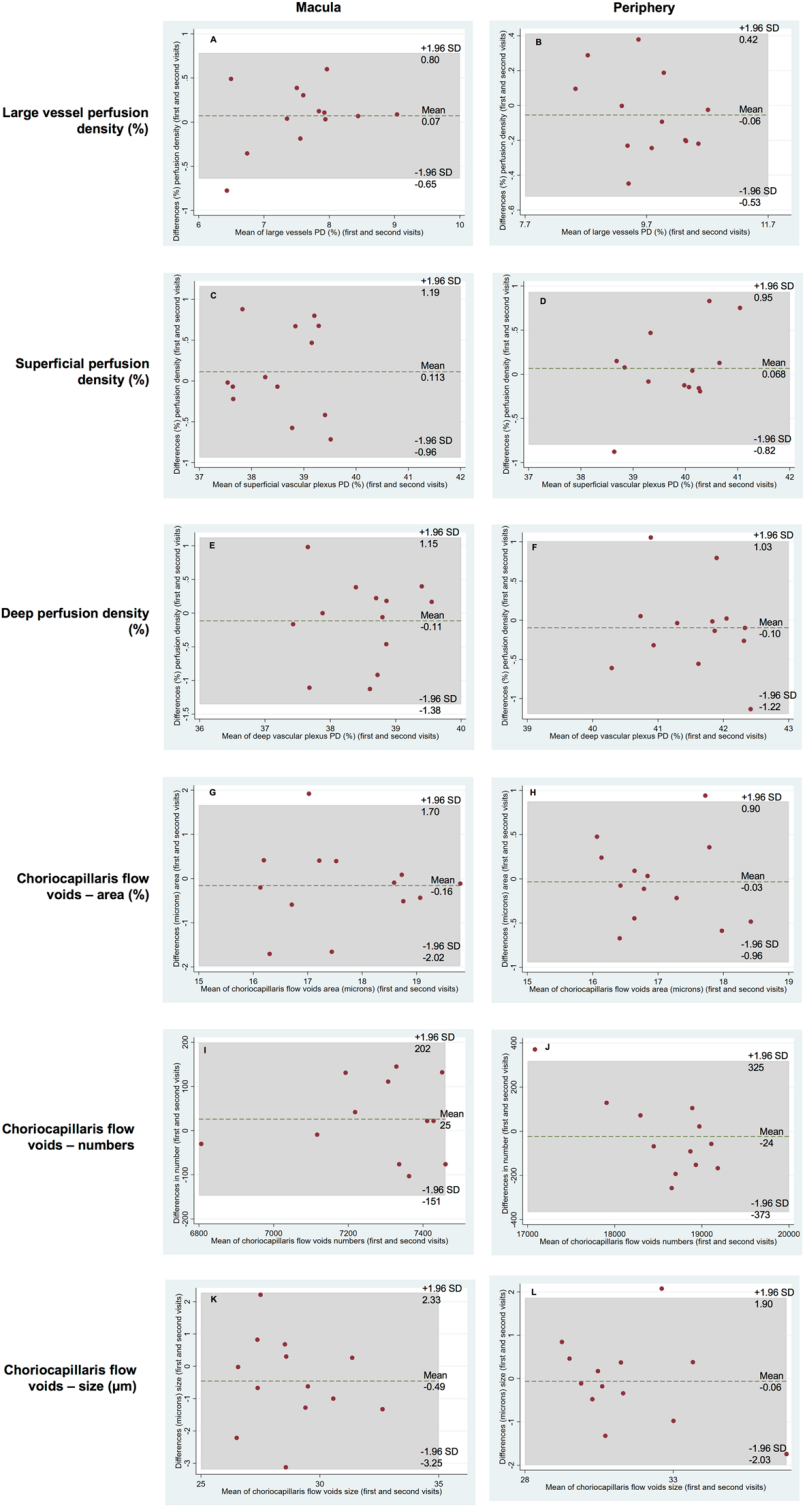
Bland–Altman plots of the different retinal and choriocapillaris flow void metrics.

Regarding the deep perfusion density obtained with the original scan (Table 1 and Fig. 3), there were no significant differences between visits in both the analysed regions (P = 0.435 for the macular region [ICC = 0.77; 95% limits of agreement, − 1.38% to 1.15%], and P = 0.456 for the peripheral region [ICC = 0.84; 95% limits of agreement, − 1.22% to 1.03%], respectively), suggesting good repeatability. The mean differences in macular and peripheral deep PD between visits were − 0.11% and − 0.10%, respectively (Fig. 4E,F). The spread of the differences remained consistent across the range of PD measurements.

The 3 filters presented with different measurements in both regions (25.16 to 33.24% for the macular region; 34.19 to 36.09%; both P < 0.001). The ICC remained good for both regions ([ICC = 0.78 with only the Hessian filter for the macular region and ICC = 0.78 to 0.88 with any of the three filters for the peripheral region). The ICC for the macular region dropped when the Gabor and Bayesian filters were used (ICC = 0.21 to 0.70; Table 1). All three filters achieved good correlation with the original scan for the macular regions (r = 0.66 to 0.79). For the peripheral region, the Hessian and Bayesian filters (r = 0.70 to 0.77) achieved moderate to good correlation with the original scan whereas the correlation was poor with the Gabor filter (r = 0.48; P = 0.081).

### Choriocapillaris flow voids

**Figure 5:**
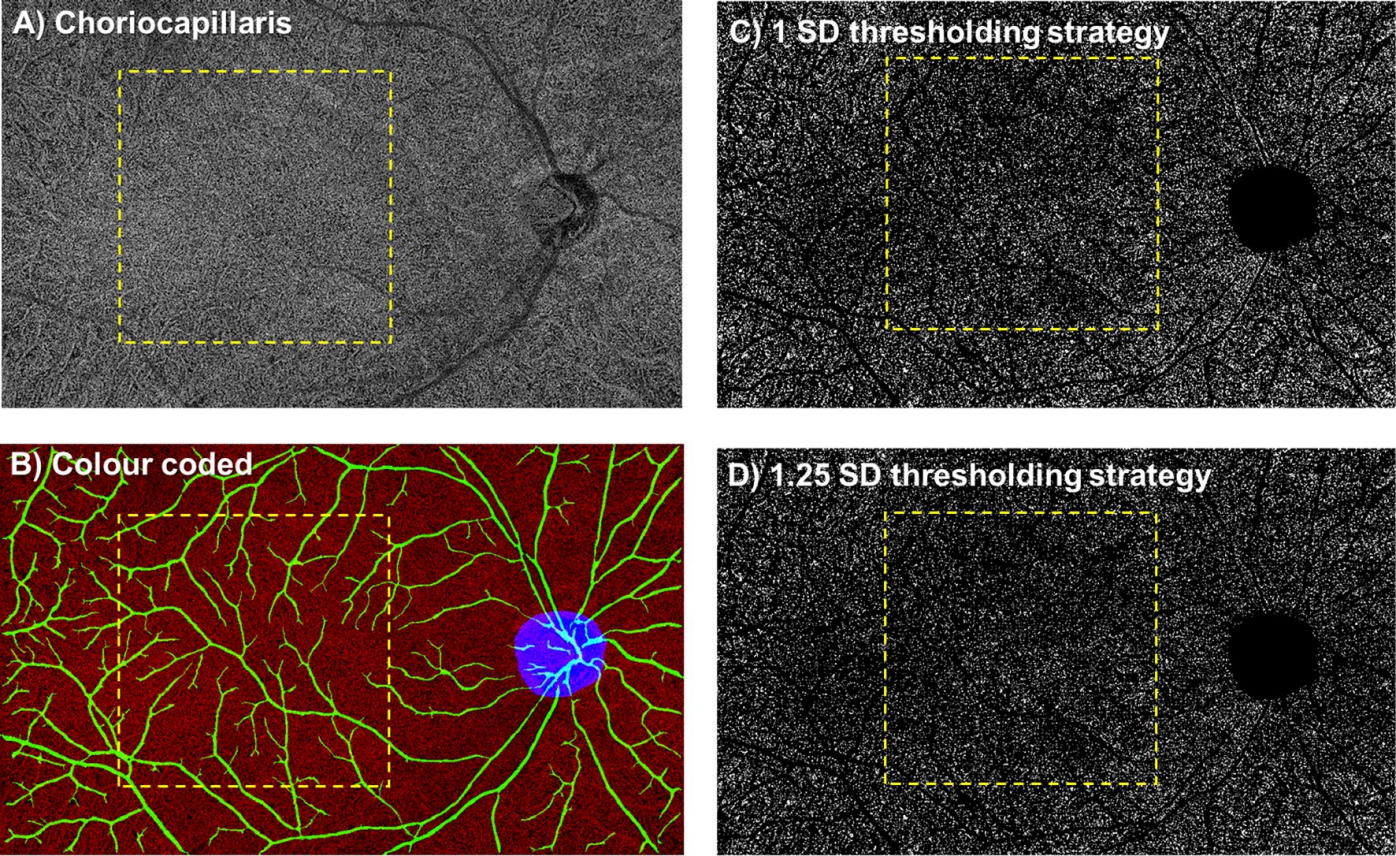
The choriocapillaris image (**A**) was processed to obtain the choriocapillaris flow void metrics using either (**C**) 1 standard deviation (SD) or (**D**) 1.25 SD thresholding algorithms. The choriocapillaris angiogram was first inverted and then thresholded. In the colour coded image (**B**), pixels with an intensity over the applied threshold are marked in red and those pixels below the threshold are marked in black. Also, the optic disc (blue) was manually masked and large vessels (green) were automatically removed for flow void calculation. There were fewer flow voids (seen as bright white speckles) when a larger threshold was used. Flow void metrics were investigated in 2 different regions of the choriocapillaris slab: (1) the macular region (within yellow dotted box) with a 6 × 6-mm^2^ region centred over the fovea and (2) the peripheral region (outside the yellow dotted box). Image A was generated from the built-in review software (PLEX Elite Review Software, Carl Zeiss Meditec, Inc., Dublin, USA; Version 1.7.1.31492; https://www.zeiss.fr/content/dam/Meditec/international/ifu/documents/plex-elite/current/2660021169042_rev._a_artwork.pdf).

The choriocapillaris flow void features in terms of its area, numbers and average size of the two visits are shown in Table 2 and Fig. 5. For flow void features using the 1 SD threshold strategy, there were no significant differences between visits in both analysed regions in terms of the area (P = 0.548 for the macular region [ICC = 0.86; 95% limits of agreement, − 2.02% to 1.70%], and P = 0.369 for the peripheral region [ICC = 0.82; 95% limits of agreement, − 0.96% to 0.90%], respectively), numbers (P = 0.172 for the macular region [ICC = 0.93; 95% limits of agreement, − 151 to 202], and P = 0.792 for the peripheral region [ICC = 0.97; 95% limits of agreement, − 373 to 325], respectively) and average size (P = 0.260 for the macular region [ICC = 0.86; 95% limits of agreement, − 81 µm to 58 µm], and P = 0.393 for the peripheral region [ICC = 0.89; 95% limits of agreement, − 51 µm to 48 µm], respectively), indicating good repeatability. The macular and peripheral flow void features between visits showed that the spread of the differences remained consistent across the range of measurements (Fig. 4G–L).

**Table 2:** OCTA vascular parameters of the choriocapillaris flow voids.

Characteristics	(A) Averaged of 2 visits Mean (SD)	P value of paired t-test	(B) ICC (95% CI)	(C) Pearson’s *r* (95% CI)	P value
**Choriocapillaris flow voids—area (%)**					
Macula					
1. 1 SD	17.65 (1.28)	0.548	**0.86 (0.54, 0.96)**	Reference	
2. 1.25 SD	12.68 (0.96)	0.657	**0.87 (0.56, 0.96)**	**0.98 (0.94, 1.00)**	< 0.001
Periphery					
1. 1 SD	16.94 (0.83)	0.369	**0.82 (0.45, 0.94)**	Reference	
2. 1.25 SD	12.28 (0.76)	0.386	**0.81 (0.44, 0.94)**	**0.96 (0.88, 0.99)**	< 0.001
**Choriocapillaris flow voids—numbers**					
Macula					
1. 1 SD	7299.15 (188.01)	0.172	**0.93 (0.76, 0.98)**	Reference	
2. 1.25 SD	6793.27 (197.93)	0.78	**0.92 (0.73, 0.98)**	**0.88 (0.64, 0.96)**	< 0.001
Periphery					
1. 1 SD	18,590.43 (556.04)	0.792	**0.97 (0.92, 0.99)**	Reference	
2. 1.25 SD	17,604.18 (460.23)	0.153	**0.94 (0.82, 0.98)**	**0.73 (0.33, 0.91)**	0.003
**Choriocapillaris flow voids—size (µm**^**2**^**)**					
Macula					
1. 1 SD	720 (49)	0.26	**0.86 (0.55, 0.96)**	Reference	
2. 1.25 SD	555 (33)	0.411	**0.87 (0.58, 0.96)**	**0.98 (0.92, 0.99)**	< 0.001
Periphery					
1. 1 SD	785 (53)	0.393	**0.89 (0.68, 0.97)**	Reference	
2. 1.25 SD	600 (37)	0.546	**0.89 (0.65, 0.96)**	**0.97 (0.90, 0.99)**	< 0.001

Bold interface indicates the filter that provided good ICC (≥ 0.75) and P < 0.05 for Pearson’s correlation when compared to the 1SD thresholding strategy for each category.

*SD* standard deviation; *CI* confidence interval; *ICC* intraclass correlation coefficient.

As expected, a larger threshold resulted in fewer flow voids (P < 0.001). The ICC remained good to excellent for all flow void features in both analysed regions (ICC = 0.87 to 0.92 for the macular region and ICC = 0.81 to 0.94 for the peripheral region; Fig. 4). Values obtained with both thresholds showed good correlation for flow void features in both analysed regions (r = 0.88 to 0.98 for the macular region and r = 0.73 to 0.97 for the peripheral region).

## Discussion

This study demonstrates that the novel widefield 15 × 9-mm^2^ scan protocol, using the SS-OCTA system, yields highly repeatable retinal and choriocapillaris vascular metrics, in both the macula and the periphery in healthy subjects. However, the set of metrics derived from the various vessel enhancement filters are not interchangeable.

### Comparison between macular and peripheral region

Further analysis of the ICC values of the retinal plexus revealed two findings. First, vascular metrics in the peripheral retina were more repeatable than those of the macula. This discovery is contrary to our hypothesis and surprising. We initially thought that the vascular parameters in the periphery would be less repeatable because of the difficulties encountered during widefield imaging, such as variations in illuminance^14^. A potential explanation is the difference in study population. Since our patients are younger, their scans may be less susceptible to poor illumination caused by poorer fixation, smaller pupils, and media opacities (e.g. cataracts). Another possible explanation can be attributed to the spatial distribution of the vessels. Peripheral vessels tend to be sparser and hence easier to capture than vessels at the macular region^21^. Additionally, the proportion of large vessels occupying the periphery is higher (9.74% vs 7.72%) and larger vessels are easier to segment, leading to more repeatable metrics. This result suggests that perfusion density measurement of the peripheral region is more reliable when assessing the 15 × 9-mm^2^ scans. Given that peripheral vascular changes occurred in diabetics^8^ and that OCTA abnormalities precede clinically detectable changes in patients with diabetes^22^ and hypertension^23,24^, this finding further highlights the potential role of widefield OCTA as a screening tool.

### Comparison between superficial and deep plexuses

The repeatability of superficial perfusion density, particularly for the 15 × 9-mm^2^ OCTA scan protocol has not been studied. An earlier study used a 12 × 12-mm^2^ OCTA scan and showed a good ICC of 0.81 for retinal density measurements, which was comparable to our study^14^. Another study found moderate ICC of 0.66 for superficial vessel density when using five montaged 12 × 12-mm^2^ scans. The lower ICC with montaged OCTA may be explained by the fact that widefield montaged OCTA scans are more prone to noise and artefacts, such as vignetting^16^. Future studies can assess the repeatability of retinal vascular metrics of montaged two 15 × 9-mm^2^ scan, which covers up to 50° of the posterior pole.

The ICC values of the perfusion density of the DCP are generally lower than those of the SCP. This difference may be because of the difficulty of visualising the deep retinal plexus, due to a denser capillary network and projection artefacts. SCP contains both large vessels as well as capillaries while the DCP contains finer capillaries, which are harder to capture^4^. Projection artefacts in the DCP are a result of the high scattering dynamics of blood within the vessels of the SCP, creating artefacts that interfere with the interpretation of retinal angiographic results^25^. Despite using the projection removal function in the review software, residual project artefacts still exist, with the outlines of the large retinal vessels still visible (Fig. 3). Hence, when performing quantitative analysis, studying the superficial vascular network can be more reliable.

### Vessel enhancement filters

We also evaluated the impact of different vessel enhancement filters on the repeatability of OCTA vascular parameters of the retinal plexuses. While metrics from the original scan were highly repeatable for the SCP, use of the Hessian and Gabor filters further improved the ICC values. However, the various filters yielded differing vessel density measurements. For example, the PD of the SCP reduced significantly after Gabor filtering (24.12% vs 38.57%). These differences likely arose due to the inherent properties of the enhancements and detection of capillaries. Images processed using the Gabor filter failed to pick up finer capillary structures while Hessian and Bayesian filters preserved most of the capillaries observed in the original scan (Fig. 2). Given the importance of imaging the microvasculature in view of its higher susceptibility to damage in DR^26^, the Hessian filter is likely more suitable for obtaining repeatable and reliable metrics in the SCP.

On the other hand, the original scan may be the best for calculation of vessel parameters of the DCP. Given how the DCP largely comprises of capillary networks^27^, the Gabor filter is less suitable. Compared to the original scan, use of the Hessian filter is not recommended because it yielded a lower signal-to-noise ratio as evident from its lower PD value (33.24% vs 38.50%) and the higher levels of noise at the foveal avascular zone (Fig. 3). Lastly, use of the Bayesian filter compromises the repeatability of the DCP metrics, particularly at the macula (ICC = 0.21 vs 0.70).

### Analysis of choriocapillaris flow void repeatability

The repeatability of choriocapillaris flow voids, particularly for such a wide OCTA scan protocol, has not been extensively studied. We showed highly repeatable flow void metrics and larger flow void areas in the macula compared to the periphery, which was consistent with a recent study that used montaged 12 × 12-mm^2^ SS-OCTA scans in healthy subjects^28^. There is currently no standard thresholding algorithm for flow void metrics^29–31^. It has been shown that the binarization thresholding algorithm impacted the OCTA quantitative measurements and the repeatability of the OCTA metrics^32,33^. Use of global and local adaptive binarization on OCTA images has its advantages and disadvantages. While adaptive binarization is insensitive to the illuminance unevenness over the entire field of view, it can artificially enhance the noise background of nonperfusion regions, such as the foveal avascular zone (FAZ) and the capillary dropout areas in diabetic retinopathy eyes. One key factor is the kernel size used for the adaptive threshold^34^. On the other hand, global thresholding is sensitive to the vignetting caused by the low illuminance in the retinal periphery, resulting in false nonperfusion detection. The selection of global or local thresholding method is dependent on many factors, including the imaging field of view^34^. For 12 × 12-mm^2^ field of view, peripheral illuminance unevenness is not trivial. Therefore, a local thresholding with a careful selection of the kernel size is, to our knowledge, the most appropriate. We selected a neighbourhood of 1.25 mm as the window size, which is a balance between supressing the background noise within the FAZ and compensating the illuminance unevenness. As evidenced by the lower flow void values obtained from the 1.25 SD thresholding strategy, it is crucial to document and assess patients longitudinally with a consistent method.

Even though the ICC is relatively good for flow void metrics (ICC > 0.81), the limits of agreement indicated a large variance particularly for the number of flow voids. Between visits, the 95% limits of agreement for the numbers of flow voids in the macular region varied from − 151 to 202 (Fig. 4I,J). In healthy subjects, the flow voids tend to be smaller and more numerous^35^ and hence are more difficult to spatially correlate between scans. This challenge is mainly due to insufficient sampling and lateral resolution of the widefield OCTA system to capture individual flow void in healthy subjects. While this lack of uniformity may be an issue in healthy subjects, variations in flow void assessment using widefield OCTA may be less problematic for diseased eyes. This is because flow voids are significantly larger and fewer in numbers in eyes with ocular diseases, making them easier to correlate between visits^29,36,37^.

### Limitations

This study has a few limitations. The cohort size was small and of healthy and young individuals, which may not be generalisable to older patients and individuals with diseased eyes, where reduced reproducibility has been reported for 3 × 3 OCTA scans^38^. Widefield 15 × 9-mm^2^ scanning protocol was introduced recently and the repeatability study in older patients free from eye diseases is ongoing. A widefield OCTA scan typically requires a longer duration to complete, which may be more challenging for patients who are elderly or with macular diseases who may suffer from poorer fixation and could further reduce the repeatability of the exam. Enabling the inbuilt eye tracking program could mitigate this issue but would take an even longer time to scan^39^. Additionally, eyes with disease could be prone to segmentation errors and confound the accuracy of capillary density measurements. The occurrence of segmentation errors increased substantially in diseased eyes^25^. Ghasemi Falavarjani, et al.^40^ reported that the segmentation errors of retinal layers further led to capillary density measurement errors, which occurred in all eyes with diabetic macular oedema and one-third of healthy eyes. Further studies involving older patients and diseased eyes should be able to better address these concerns.

## Conclusion

In conclusion, our analysis demonstrated good repeatability of widefield SS-OCTA derived retinal perfusion density measurements and choriocapillaris flow void features in healthy individuals using a prototype OCTA software. In addition, vessel enhancement filters impacted the repeatability of retinal density measurements especially for the macular region. Measurements from different vessel enhancement filters were not comparable. These findings highlight the importance of explicitly stating the post-image processing methodology in OCTA studies.

## Materials and methods

### Study participants

We conducted a prospective observational study where 15 healthy individuals were enrolled from the Singapore Eye Research Institute, Singapore National Eye Centre, Singapore. Healthy individuals aged between 22 and 47 years old with no history of ocular or systemic problems were included. We excluded anyone with vision complaints and history of any surgical intervention (including refractive surgery). Ethics approval was obtained from the SingHealth Centralized Institutional Review Board. Written, informed consent was obtained for all participants in adherence to the Declaration of Helsinki.

Each participant underwent assessments that included blood pressure measurements, autorefraction, ocular biometry, retinal photography, and OCTA. Previous reports did not find any significant difference in OCTA metrics between fellow eyes in healthy participants^41,42^. Both eyes were evaluated but only the right eye was included for analysis unless its image quality was inadequate. In such instances, the left eye was studied instead. Posterior pole evaluation was attempted without pupillary dilatation but if needed, pupils were dilated with 1% tropicamide and 2.5% phenylephrine hydrochloride. In the current study, all our subjects were young (31 ± 6 years old) and cooperative (most are research staff), hence we did not have to perform pharmacological pupil dilation.

### Image acquisition

All OCTA scans were performed by a single experienced technician using the PLEX Elite 9000 (Carl Zeiss Meditec, Inc., Dublin, USA; Version 1.7), a SS-OCTA system which features a central wavelength of 1050 nm, a speed of 100,000 A-scan per second and an axial resolution of 6.3 µm and transverse resolution of 20 µm in tissue. The FastTrac motion correction software, based on a line scan ophthalmoscope, was enabled to minimize motion artefacts. Each participant received a 15 × 9-mm^2^ scan, with each volumetric data consisting of an isotropic sampling (800 × 534) and two consecutive B-scans obtained at each raster location to derive the angiographic information using an optical microangiography protocol^43^. The signal strength index is provided by the PLEX Elite 9000 system, which indicates scan quality, is measured on a scale of 1 to 10, where 1 indicates poor image quality and 10 indicates best image quality We selected a signal strength of > 8 to indicate acceptable image quality^44,45^. Poor-quality images (signal strength index < 8, significant motion artefact, misalignment or incorrect segmentation) were excluded and scans was repeated if necessary. All participants underwent second OCTA measurements one week later.

Each scan was automatically segmented, generating the superficial (SCP) and deep capillary plexus (DCP) as well as the choriocapillaris by the PLEX Elite Review Software Version 1.7. The SCP spans the inner limiting membrane (ILM) to the inner plexiform layer (IPL), while the DCP spans the IPL to the outer plexiform layer (OPL). The choriocapillaris was taken from 31 to 40 µm below the retinal pigment epithelium (RPE) layer. Images were checked to ensure correct segmentation by the automated instrument software and no manual adjustment was needed.

### Image processing

**Figure 6:**
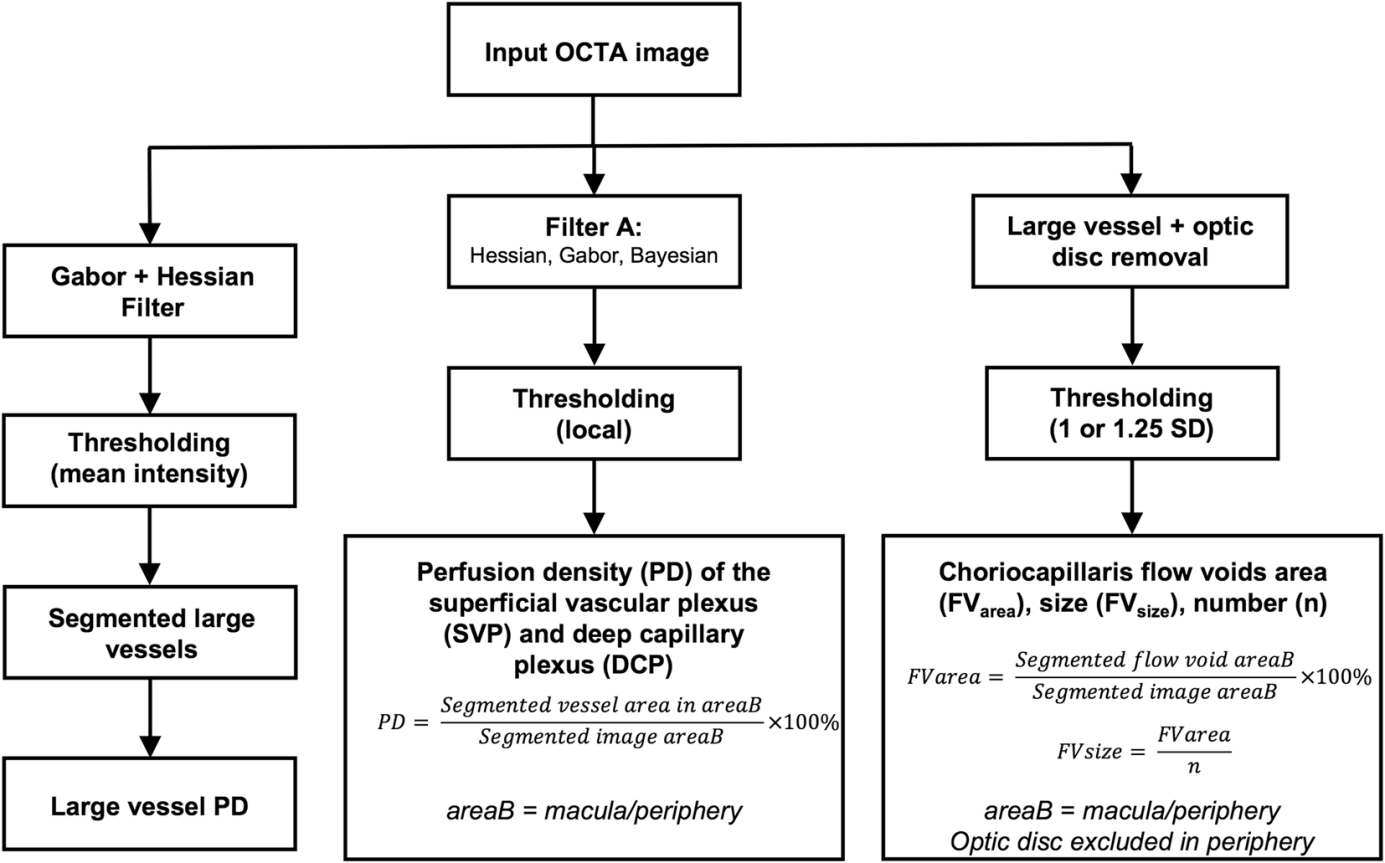
Flowchart of post-imaging processing to obtain perfusion densities of the (1) large vessels, (2) superficial and deep capillary plexuses as well as (3) choriocapillaris flow void area, number, and size.

OCTA images of the superficial and deep retinal plexus as well as the choriocapillaris were exported for post-image processing in MATLAB (The MathWorks Inc., Natick, MA). To obtain the large vessel perfusion densities (PD), calculated as the ratio of the segmented large vessels over the total image area (Fig. 1), a combined Gabor and Hessian filter was applied to increase vessel contrast and the large vessels were segmented under a derived threshold from the mean intensity^8^ (Fig. 6). For retinal plexus analysis, filters were applied to enhance vascular structures (Figs. 2, 3). A total of 3 filters were applied and compared: (1) a Hessian filter (size range = [15–44] µm)^17,18,46^; (2) a Gabor filter (wavelength = 20π, orientation interval: π/16)^19,20^; and (3) a modified Bayesian residual transform-based filter^47^. The images were then binarized by local adaptive thresholding^48^. We selected a neighbourhood of 1.25 mm as the window size to obtain the PD, defined as the ratio of the segmented vessel area over the total image area (Fig. 6).

To derive choriocapillaris flow void metrics, the large vessels were removed with the above method and the optic disc was manually masked. Choriocapillaris flow voids indicate areas of decreased choriocapillaris perfusion^30^. An increase in the density of flow voids is indicative of choroidal capillary dropout and may be associated with restricted supply of the outer retina and development and progression of photoreceptor loss^49^. Flow voids were then calculated using either 1 or 1.25 standard deviation (SD) thresholding algorithms based on previous publications (Fig. 6)^30,31^. Flow void metrics, including total flow void area (i.e. flow void area per unit area), number of flow voids, and size of each flow void (i.e. average area of each flow void), were calculated (Fig. 5). Angiographic data from the 15 × 9-mm^2^ scans were further divided into 2 regions: the macular region, which was defined as a 6 × 6-mm^2^ region centred over the fovea, and the peripheral region, which was assessed as all regions outside the central 6 × 6-mm^2^ (Figs. 2, 3 and 5).

### Statistical analysis

Dependent t-test was used to assess the differences between paired measurements obtained at the two different visits. Independent t-test was used to assess the differences between measurements obtained with different filters. The inter-visit repeatability of the OCTA metrics was investigated using intraclass correlation coefficients (ICC), where values < 0.50, 0.50–0.75, 0.75–0.90 and > 0.90 indicated poor, moderate, good and excellent repeatability, respectively^50^. We then examined the correlation between metrics derived from filtered OCTA scans and original scans (i.e. no filter applied) using Pearson’s *r*. This is important because a poor vessel enhancement filter can still show excellent ICC by increasing its noise level until saturation. For such cases, the resultant scan will be poorly correlated with the original scan. Statistical analyses were performed using Stata version 16.0 (StataCorp LLC, College Station, TX).

## Data Availability

The datasets generated during and/or analyzed during the current study are not publicly available due to the terms of consent to which the participants agreed but are available from the corresponding author on reasonable request.
